# The impact of humor therapy on people suffering from depression or anxiety: An integrative literature review

**DOI:** 10.1002/brb3.3108

**Published:** 2023-06-21

**Authors:** Xuefeng Sun, Jindan Zhang, Yidan Wang, Xiaotu Zhang, Sixuan Li, Zihan Qu, Hongshi Zhang

**Affiliations:** ^1^ School of Nursing Changchun University of Chinese Medicine, Jingyue National High‐Tech Industrial Development District China

**Keywords:** anxiety, depression, humor therapy, laughter therapy, psychosocial intervention

## Abstract

**Objectives:**

To identify and synthesize existing research on the effectiveness and feasibility of multiform humor therapy on people suffering from depression or anxiety, with the hope of benefiting future research.

**Methods:**

An integrative literature review of quantitative, qualitative, and mixed studies was performed. The PubMed, Cochrane Library, Web of Science, Embase, and CINAHL databases were searched up to March 2022. Two independent reviewers conducted each stage of the review process, by assessing eligibility using preferred reporting items for systematic review and meta‐analyses (PRISMA) and quality appraisal using the Mixed Methods Appraisal Tool, and data extraction.

**Results:**

In this integrative review, 29 papers were included, containing 2964 participants across a diverse range of studies, including quantitative, qualitative, and mixed methods. The articles were from the United States, Australia, Italy, Turkey, South Korea, Iran, Israel, China, and Germany. The findings indicated that most of the subjects thought humor therapy was effective in improving depression and anxiety while a few participants considered the effect insignificant. However, more high‐quality studies will be needed to confirm these conclusions.

**Discussion:**

This review collated and summarized findings from studies examining the impact of humor therapy (medical clowns, laughter therapy/yoga) on people with depression or anxiety, including children undergoing surgery or anesthesia, older people in nursing homes, patients with Parkinson's disease, cancer, mental illness, and undergoing dialysis, retired women, and college students. The results from this review may help inform future research, policy, and practice in humor therapy to improve people's symptoms of depression and anxiety.

**Impact:**

This systematic review objectively evaluated the effect of humor therapy on depression and anxiety. As a simple and feasible complementary alternative therapy, humor therapy may provide a favorable alternative for clinicians, nurses, and patients in the future.

## Graphical abstract

This systematic literature review objectively evaluates the effect of humor therapy on depression and anxiety. As a simple and feasible complementary alternative therapy, humor therapy provides favorable conditions for clinicians, nurses and patients in the future.

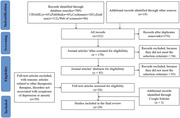

## 1 INTRODUCTION

Depression and anxiety are common mental disorders and leading causes of disability worldwide (Li et al., 2022). Based on 2021 data (Abeysekera & De Zoysa, 2021; Chan et al., 2022), the worldwide prevalence of depression and anxiety per 100,000 persons was found to be 3153 and 4802 cases, respectively. Of these, 15–20% of children and adolescents also suffered from anxiety and depression. The recent COVID‐19 pandemic had also significantly increased the prevalence of depression and anxiety disorders (Chan et al., 2022). The typical symptoms of depression include low mood, decreased interest, memory loss, slow thinking, decreased volitional activity, sleep disturbances, loss of appetite, and suicidal thoughts. In addition to rapid heart rate, weakness, fatigue, and other physiological reactions, the primary symptom in patients with anxiety disorders is the psychological experience and feeling of excessive worry. The Diagnostic and Statistical Manual of Mental Disorders Fifth Edition (DSM‐5) details depression and anxiety disorders and their typical symptoms. There is a category of depressive and anxiety symptoms that do not fit with the other diagnoses, which the DSM‐5 calls unspecified depressive disorders and unspecified anxiety disorders. These can include children with significant mood changes before surgery and depression or anxiety due to illnesses such as cancer and hemodialysis.

Anxiety and depression are frequently comorbid in psychiatry and are influenced by various factors such as gender, socioeconomic status, and social support (Bandelow et al., 2017). In recent years, depression and anxiety have become more prevalent in younger people, yet many patients are still not receiving adequate treatment. Therefore, it is essential to explore positive psychosocial interventions (Dubovsky, 2021).

Although psychiatric medications often alleviate symptoms, they can be costly and have severe side effects, making them unsuitable for elderly or pediatric patients. Some healthcare professionals believed medication was not feasible for these patients (Ebrahimi et al., 2022). Consequently, nonpharmacological approaches have been proposed to address these concerns. Complementary and alternative medicine (CAM) is increasingly used for treating various diseases, including psychiatric disorders such as depression and anxiety. CAM therapies are classified into four categories, namely, biologically based therapies, manipulative and body‐based therapies, mind‐body therapies, and alternative medical systems (Trkulja & Barić, 2020). CAM may offer a promising alternative or complementary approach to treating anxiety and depression in individuals who cannot tolerate conventional medications or prefer nonpharmacological interventions.

In recent years, humor therapy has been widely applied as a CAM. Humor therapy is defined by the Association for Applied and Therapeutic Humor as interventions that promote physical well‐being while promoting emotional, cognitive, social, or spiritual healing through the playful discovery, expression, or appreciation of absurd or incongruous situations in life (Zhao et al., 2020). This intervention helps people cope with stress, regulate emotions and promote physical and mental health (Farkas et al., 2021). According to Mallett (1995), humor therapy is a nonpharmacological intervention that improves immune function, raises discomfort thresholds, reduces stress, and protects cardiovascular and respiratory function. The contents and types of interventions used in humor therapy are varied, such as reading interesting books and cartoons, watching witty plays and comic videos, sharing anecdotes, instructing relaxation of facial muscles, laughter, meditation, encouraging singing and dancing together, role‐playing, and clown performances. Humor or laughter is primarily investigated through three theories: superiority theory, incongruity theory, and relief theory (Kuru & Kublay, 2017). Superiority theory focuses on humor, believing that people are always competing and looking for flaws in others. Laughter is a sudden realization that one is superior to others. Thus, in terms of the theory, people feel happy watching exaggerated expressions, humorous language, or funny pictures that contribute to increased self‐worth and self‐efficacy (Wilkins & Eisenbraun, 2009). Relief theory posits that laughter releases tension and depression caused by societal constraints. It suggests that individuals laugh at amusing events and subsequently feel happy and relaxed, releasing tension, anxiety, and depression, which may positively impact their physical and mental health (Ko & Youn, 2011). Lastly, Incongruity theory focuses on the process by which people understand and process humor. According to this theory, the perception of dissonance can encourage people to think positively, engage in communication and discussion, and enhance their ability to identify problems (Wilkins & Eisenbraun, 2009).

This literature review aims to find and evaluate current research on humor therapy, discussing the effectiveness and feasibility of multiform humor intervention as a psychological treatment, in the hope that this will benefit future research.

## 2 REVIEW

### 2.1 Aims

We aimed to integrate the findings of primary studies using quantitative, qualitative, and mixed methods to assess the efficacy of humor therapy in alleviating depression and anxiety. The questions addressed in this review are as follows:

Is it possible, based on baseline measurements and subsequent changes after interventions, to demonstrate that humor therapy, laughter therapy, and medical clowning can significantly ameliorate depression or anxiety in different populations?

What are the attitudes of subjects to the use of humor therapy for depression or anxiety as a complementary alternative to medication?

### 2.2 Literature search

**FIGURE 1:**
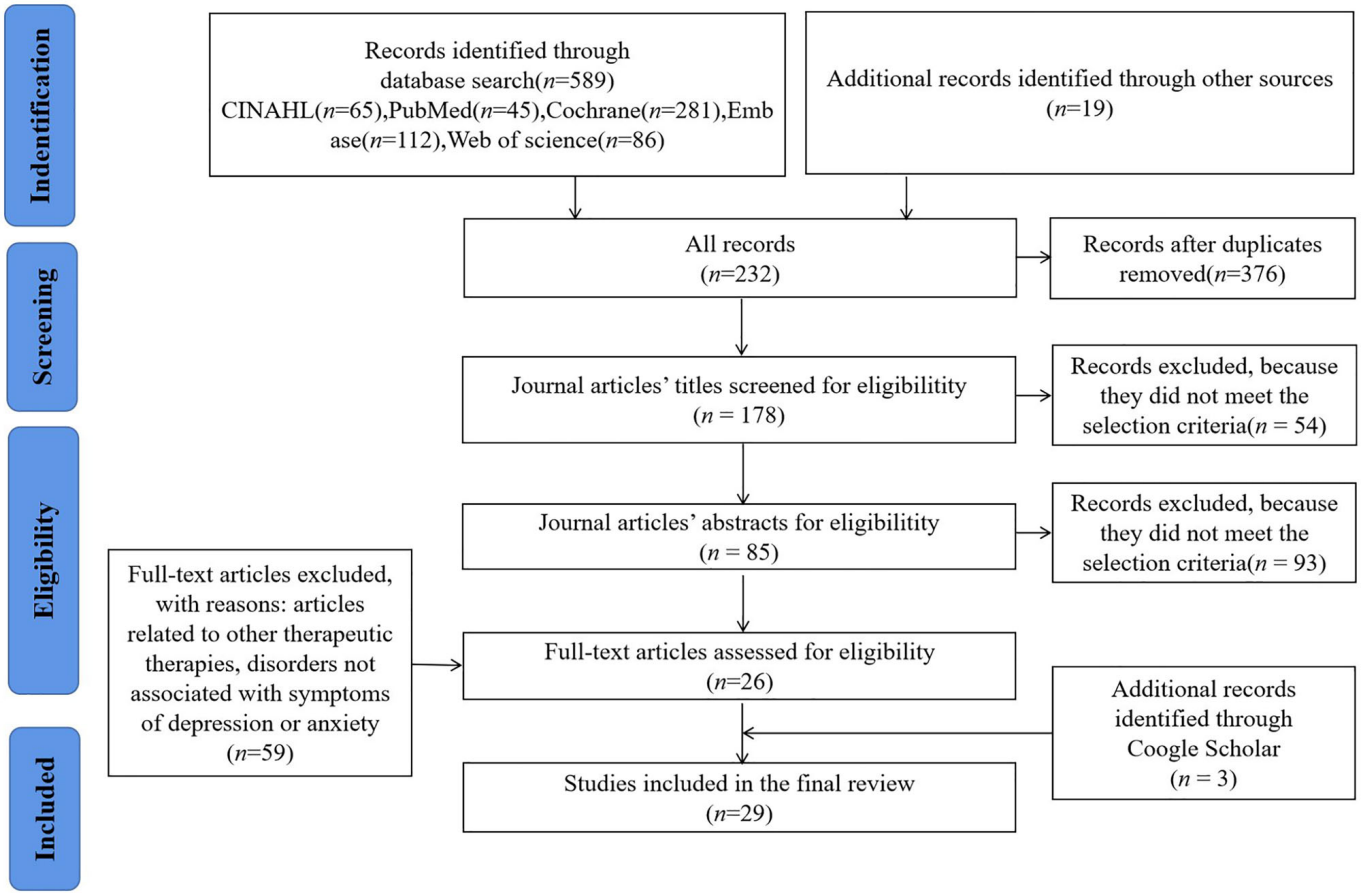
PRISMA flow diagram.

A systematic literature review was conducted with the assistance of a librarian and three master's students. The databases searched included PubMed, Cochrane, EMBASE, CINAHL, and Web of Science, and the search period spanned from May 2012 to March 2022. EndNote version X9.1 was used to screen the literature, and Table 1 provides details on the electronic databases searched, along with the syntax used. The references of the selected articles were checked multiple times. The Preferred Reporting Items for Systematic Reviews and Meta‐Analyses (PRISMA) framework was followed in this study (Moher et al., 2015), as illustrated in Figure 1.

**TABLE 1:** Search syntax for electronic databases.

Database	Syntax
PubMed	(“Laughter Therapy”[Mesh]) OR (humor therapy) OR (clown therapy) AND (“Anxiety”[Mesh]) OR/AND (“Depression”[Mesh])
Cochrane	(Laughter Therapy):ti,ab,kw OR (humor therapy):ti,ab,kw OR (clown therapy):ti,ab,kw AND anxiety:ti,ab,kw OR/AND depression:ti,ab,kw
Embase	(“humor therapy”/exp OR “humor therapy”) OR (“humor”/exp OR humor) OR (“laughter therapy”/exp OR “laughter therapy”) OR (“laughter”/exp OR laughter) OR (“clown therapy”/exp OR “clown therapy”) AND (“depression”/exp OR depression) OR/AND (“anxiety”/exp OR anxiety)
CINAHL	Laughter therapy OR humor therapy OR clown therapy AND anxiety OR/AND depression
Web of Science	ALL = (Laughter therapy) OR (humor therapy) OR (clown therapy) AND ALL = (anxiety) OR (depression)

### 2.3 Selection criteria

The inclusion criteria were the following: (1) subjects were diagnosed with an anxiety disorder or depressive disorder, according to DSM; “depressive” or “anxious” difficulties in people who did not meet the “clinical criteria,” but suffered such difficulties due to a specific situation in life (e.g., children who undergo surgery or similar), but not too extensively; (2) the article commented on humor therapy or other forms of humor intervention; (3) types of studies to include quantitative, qualitative, and mixed research; and (4) the selected studies were published in the journal or a doctoral or master's thesis; (5) written in English only. The following were excluded: (1) any of the above criteria was violated; (2) articles with animal studies; (3) the article did not discuss the effects of humor therapy on people with depression and anxiety; and (4) more than 25% of subjects’ dropouts.

### 2.4 Quality appraisal

The Mixed Method Appraisal Tool (MMAT, version 2018) was used to assess the quality of the included studies during the rigorous evaluation stage of the systematic review. MMAT can assess the methodological quality of five types of studies, namely, qualitative studies, randomized controlled trials, nonrandomized studies, quantitative descriptive studies, and mixed studies (Hong et al., 2018). After answering two screening questions for each included study, the appropriate study category was selected for evaluation and then scored according to the criteria of the selected category. Based on this, two researchers independently evaluated the methodological and five quality criteria of MMAT. Scores that met one criterion were designated “*” and scores that met all requirements were classified as “*****.” Each included study was read in detail to obtain an objective evaluation score. When the evaluation score was inconsistent or uncertain, the two people discussed it together and reached a decision. This paper did not exclude several low‐quality studies due to the objectivity of the research results. Table 3 depicts the quality scores.

### 2.5 Data extraction and synthesis

This review included 29 relevant publications, and a two‐person extraction method was used for the comprehensive extraction of vital information and data from each article. Analysis was performed using the method of Whittemore and Knafl (2005), whereby the extracted data were coded and compared to identify key concepts. Consensus between the researchers on the extracted data was reached after discussion.

## 3 RESULTS

PRISMA was used to screen the literature, resulting in a total of 29 articles included in this study (see Figure 1). The searches yielded a total of 608 studies, 376 of which were duplicated and, therefore, removed. The remaining 232 studies were chosen based on titles and abstracts. One hundred and forty‐seven studies were not included in the scope of the review, which led to the retrieval of 85 studies for possible inclusion. Full‐text retrievals were evaluated against inclusion criteria. Fifty‐nine studies did not meet the inclusion criteria for various reasons, for example, the study was about disorders not associated with symptoms of depression or anxiety, and were excluded. The study characteristics, including author, region, MMAT quality ratings, population type, study design, intervention group content, and control group content, can be investigated in Table 2. Table 3 showed the evaluation tools, baseline, outcome data, and attitude in each study.

**TABLE 2:** Characteristics and analytics of included studies (*N* = 29).

Author	MMAT	Country	Population type	Study design	Intervention group	Control group
(Meiri et al., 2016)	MMAT****	Israel	Children **(2−10 years)**	RCT	**Clown intervention: *(30 children)***	Anesthetic cream (EMLA) ***(30 children)*** Neither clown nor EMLA ***(30 children)***
(Dionigi et al., 2014)	MMAT****	Italy	Children **(2−12 years)**	RCT	**Clown intervention: *(52 children)***	Routine intervention ***(25 children)***
(Liguori et al., 2016)	MMAT****	Italy	Children **(6−11 years)**	RCT	**Clown intervention: *(20 children)*** A 6‐min video of two clown doctors providing operating room information for children to watch before surgery.	The standard informative Intervention ***(20 children)***
(Goldberg et al., 2014)	MMAT*****	Israel	Children **(8−17 years)**	RCT	**Clown intervention: *(45 children)*** Clowns accompany children for skin allergy tests.	Routine operation ***(46 children)***
(Felluga et al., 2016)	MMAT****	Italy	Children **(4−11 years)**	RCT	**Clown intervention: *(20 children)*** Accompanied by parents and clowns, clowns perform various performance	Only parents accompany their children ***(20 children)***
(Kocherov et al., 2016)	MMAT****	USA	Children **(2−16 years)**	RCT	**Clown intervention: *(40 children)*** In a clown group, children interacted with clowns before entering the operating room and stayed with them and their parents throughout the anesthesia induction process.	In the control group, children were accompanied only by a parent to the operating room. ***(40 children)***
(Tener et al., 2016)	MMAT*****	Israel	Children **(5−16 years)**	Qualitative study	**Clown intervention: (*9 children and their parents*)** **In‐depth semistructured interview**: The child and the clown were constructed in five clear stages during the medical encounter: (1) the first moment of the meeting; (2) the preexamination period; (3) the medical examination; (4) the postexamination period; and (5) parting. Then we interviewed the participants, and they described the significance of the clown retrospectively and how it clown projected onto their perception of the hospital, the examination, and their narrative.	
(Agostini et al., 2014)	MMAT***	Italy	Children **(3−12 years)**	RCT	**Clown intervention: *(25 children)*** The clowns entered the waiting room and started playing with the child, staying with him or her for about 30 min.	**Control group** with a routine procedure, in which children and their mothers stayed in the waiting room without clowns for the same period as the intervention group. ***(25 children)***
(Kurudirek et al., 2021)	MMAT***	Turkey	Children **(7−12 years)**	RCT	**Clown intervention: *(83 children)*** Professional clowns (colorful clothes, clown makeup, juggling, funny voices of animals, puppets, whistling, funny songs, jokes, exercise, drawing pictures, video distraction techniques) were used.	A standard blood sampling procedure was administered to the children included in the **control group** without any intervention ***(83 children)***
(Cai et al., 2014)	MMAT***	China	People with mental health conditions	RCT	**Humor therapy: *(15 patients)*** Humor skill training: the course begins with an opening fun activity, followed by group discussions, group games or practical applications, followed by humorous appreciation activities, and finally practicing “home‐play.” 45−60 min in each session for 2 sessions per week for 5 weeks.	Doing handwork ***(15 patients)***
(Rudnick et al., 2014)	MMAT****	USA	People with mental health conditions	Mixed methods	**Humor therapy**: **The experimental arm: *(12 patients)*** Standup comedy training **The active control arm: *(13 patients)*** Discussing comedy video The duration of intervention was 3 h per week for 3 months.	Routine training ***(11 patients)***
(Tagalidou et al., 2019)	MMAT****	German	People with mental health conditions	RCT	**Humor therapy*: (19 subjects)*** In detail, our manual consisted of seven sessions held once a week for 90 min. Each session dealt with a specific topic. The sessions generally contained psychoeducational elements and exercises such as role‐plays and games to train humor. Between sessions, participants had to do homework to practice what they had learned.	Received usual care ***(18 subjects)***
(Bressington et al., 2019)	MMAT****	China	Depression **(18−60 years)**	Mixed methods	**Laughter therapy: *(23 patients)*** The four steps of LT are composed of (1) warm‐up exercises, (2) deep breathing exercises, (3) childlike playfulness, and (4) laughter exercises.LT intervention consisting of eight sessions over 4 weeks.	Treatment‐as‐usual ***(27 patients)***
(Shahidi et al., 2011)	MMAT***	Iran	Depression **(60−80 years)**	RCT	**Laughter therapy: *(20 women)*** Laughter exercises are interspersed with deep breathing to bring physical and mental relaxation. **Exercise therapy: *(20 women)*** Ten sessions of an aerobic group exercise program including jogging and stretching exercises were used.	Routine daily activities ***(20 women)***
(Kim et al., 2015)	MMAT****	South Korea	Cancer patients	RCT	**Laughter therapy: *(33 patients)*** Sessions began with a 10‐min introduction on the effect of laughter followed by 40 min of patient participation in physical activities to make them laugh aloud. Three laughter therapy sessions lasting 60 min each.	Regular treatment ***(29 patients)***
(Lee et al., 2020)	MMAT*****	South Korea	Cancer patients (≥**18 years)**	Quantitative nonrandomized	**Laughter therapy: *(17 patients)*** The 8‐week laughter program included a weekly 60‐min group session composed of laughter, deep breathing, stretching, meditation, and entrainment music‐related activities (body movement, dancing).	Regular treatment ***(19 patients)***
(Genc & Saritas, 2020)	MMAT****	Turkey	Cancer patients (≥**18 years)**	RCT	**Humor therapy: *(44 patients)*** Patients were asked to watch nursing intervention materials consisting of a 10‐min excerpt from a classic Turkish comedy film on a tablet computer.	Received no intervention ***(44 patients)***
(Bega et al., 2017)	MMAT****	USA	Parkinson's patients	RCT	**Laughter therapy: *(11 patients)*** One hour improvisation theater sessions led by The Second City® faculty took place once a week for 12 weeks.	Regular treatment ***(11 patients)***
(Benn et al., 2020)	MMAT****	USA	Hemodialysis patients	RCT	**Laughter therapy: *(72 subjects)*** The intervention group received a once weekly, 30‐min group laughter therapy session for 8 weeks. Each 30‐min session consisted of breathing and stretching exercises; facilitated by intentional laughter exercises; and finished with laughter meditation.	Receiving dialysis or usual care ***(79 subjects)***
(Armat et al., 2022 )	MMAT****	Iran	Retired women **(50−70 years)**	RCT	**Laughter therapy: *(33 women)*** Laughter yoga exercises included appreciation laughter, deep yoga breathing, woodchopper pose, stretching exercises with ball and silent laughter, clapping with song, argument laughter while walking in the imaginary forest, and meditation exercises. The interventions group received LY twice weekly for 8 weeks.	Routine daily activities ***(29 patients)***
(Ko et al., 2022 )	MMAT*****	South Korea	Married immigrant women	RCT	**Laughter therapy: *(19 women)*** We included various activities along with simulated laughter, such as clapping, singing, dancing, and playing games. The intervention group participants were given LT twice a week for 2 weeks.	The participants in the waiting‐list control group received no treatment during the same period but received it afterward. ***(22 women)***
(Kiyak & Kocoglu, 2021)	MMAT****	Turkey	Infertile women	RCT	**Laughter therapy: *(71 women)*** Laughter therapy was applied for 15−20 min. Then, the lights were turned off and progressive muscle relaxation exercises were performed for 15−20 min under candlelight and accompanied by music. The intervention group received progressive muscle relaxation and laughter therapy for 40 min in each session for 3−4 sessions.	Received routine care: includes the nurse's evaluation of the in The fertile couple, counseling, and arranging a psychologist. ***(70 women)***
(Ko & Youn, 2011)	MMAT***	South Korea	Elderly (≥**65 years)**	RCT	**Laughter therapy: *(48 subjects)*** Explained the effects of laughter and showed a video of practical laughter therapy that the participants could understand easily. Then, they were directed to relax their facial muscles, clap hands, and say hello to each other. The subjects in the LT group undergo LT four times over 1 month.	Routine daily activities ***(61 subjects)***
(Heidari et al., 2020)	MMAT*****	Iran	Elderly (≥**60 years)**	RCT	**Laughter therapy: *(45 subjects)*** Intervention in each session was performed in modes of playing musical and visual slides and humorous video clips (30 min), as well as holding happy and joyous games with prizes of humor telling (15 min) and joke telling (15 min). Ten sessions of LT were administered to the intervention group subjects 3 times a week.	Routine daily activities ***(45 subjects)***
(Ghodsbin et al., 2015)	MMAT****	Iran	Elderly (≥**60 years)**	RCT	**Laughter therapy: *(36 subjects)*** The experimental group participants attended a laughter therapy program consisting of two 90‐min sessions per week lasting for 6 weeks. The program included performing breathing and physical exercises as well as laughter techniques.	Received no intervention ***(36 subjects)***
(Low et al., 2014)	MMAT*****	Australia	Elderly (≥**50 years)**	RCT	**Humor therapy: *(189 subjects)*** Professional “Elder Clowns” provided 9–12 weekly humor therapy sessions, augmented by resident engagement by trained staff “Laughter Bosses.”	Received usual care ***(209 subjects)***
(Brodaty et al., 2014)	MMAT***	Australia	Elderly (≥**50 years)**	RCT	**Humor therapy: *(189 subjects)*** Professional performers called “Elder Clowns” provided 9–12 weekly humor therapy 2‐h sessions, augmented by trained staff, called “Laughter Bosses.” Humor therapy was conducted over 12 weeks.	Received usual care ***(209 subjects)***
(Low et al., 2013 )	MMAT*****	Australia	Elderly (≥**50 years)**	RCT	**Humor therapy: *(189 subjects)*** Elder Clowns tailored their interactions to maximize resident engagement, laughter and enjoyment, adapting to the residents’ background, personality, mood, and physical and cognitive abilities.	Received usual care ***(209 subjects)***
(Ozturk & Tezel, 2021)	MMAT****	Turkey	A first‐year student	RCT	**Laughter therapy: *(36 subjects)*** The intervention group took eight sessions of laughter yoga, two weekly sessions for 4 weeks. Each laughter yoga session lasted about 40−45 min. **Part 1**: Deep breathing exercises (5–10 min) **Part 2**: Warm‐up exercises (10 min) **Part 3**: Childlike playfulness (10 min) **Part 4**: Laughter exercises (15 min).	Received rout ***(36 subjects)***

**TABLE 3:** Baseline and outcomes of included study (*N* = 29).

Author	Item	Evaluation tool	Baseline	Outcome	Attitude
(Meiri et al., 2016)	Anxiety	VAS	The mean number of blood examinations: **Intervention group**: 2.48 ± 1.80 **EMLA group**: 2.88 ± 2.63 **Control group**: 1.90 ± 1.34 There were no differences between the groups, and there was a generally significant negative correlation between the number of previous blood exams and anxiety in the current exam (*r* = −0.25, *p* = .012).	The intervention group was significantly lower with clown than in the control group or EMLA (2.6 ± 1.2 vs. 3.7 ± 1.3 or 3.8 ± 1.6, ***p* < .01** for both).	Positive
(Dionigi et al., 2014)	Anxiety	m‐YPAS	**Intervention group**: 50 (23−97) **Control group**: 33 (23−97)	**Intervention group**: 33 (23−83) **Control group**: 43 (23−100) **Pre‐post = 0.002; group = 0.004** (***p* < .01**)	Positive
(Liguori et al., 2016)	Anxiety	m‐YPAS	The **initial mean** (SD) m‐YPAS scores were 37.3 (21.7) and 37.1 (13.8) for the experimental and control groups, respectively.	The mean (SD) difference between the m‐YPAS score at the first and second measurements of each participant was −2.8 (7.2) in the experimental group and 10.7 (10.8) in the control group. The 13.5‐point difference between these averages was statistically significant (***p* = .003**).	Positive
(Goldberg et al., 2014)	Anxiety	STAI	**Intervention group**: 30.3 ± 5.4 **Control group**: 33.6 ± 5.7 *p* = .1	A significant reduction in state‐STAI was found in the clowns group (27.1 ± 4.2), when compared with the regular group (34.3 ± 7.6), ***p* = .002, *p* < .05**.	Positive
(Felluga et al., 2016)	Anxiety	CAPS	**Intervention group**: 2 (1−3) **Control group**: 2 (0−3) *p* = .759	Anxiety during the medical care, a significant reduction in CAPS was found in the clowns group (1 (0−2)), when compared with the control group (2 (0−3)), ***p* = .013, *p* < .05**.	Positive
(Kocherov et al., 2016)	Anxiety	m‐YPAS	There was no difference between the children's ages in both groups (*p* = .732).	The patients from the intervention group demonstrated a lower preoperative anxiety index upon (***p* = .0319**) and after surgery (***p* = .0042**)	Positive
(Tener et al., 2016 )	Anxiety	Using an in‐depth semistructured interview guides, one for the parent and another for the child.	A purposive sample of nine children, six undergoing an endoscopic examination and three an anogenital examination and their accompanying parents (six mothers and three fathers) were invited to participate in **in‐depth interviews**. The Children's ages ranged between 5 and 16 (average 9.7 years).	The study indicates that with a medical clown, the anogenital examination and the whole medical encounter are perceived not only as less frightening and less distressing by the child and family, but may even become a positive empowering experience, shaping perceptions toward the hospitalization experience, as well as the life narrative.	Positive
(Agostini et al., 2014)	Anxiety	STAI(Y‐I)	Waiting room: **Intervention group**: 43.76 ± 11.45 **Control group**: 46.04 ± 11.67	After the separation: the results showed that maternal state anxiety scores significantly changed over time. (***p* = .0001**) **Intervention group**: 35.36 ± 8.96 **Control group**: 38.44 ± 7.37	Positive
(Kurudirek et al., 2021)	Anxiety	CFS	**CFS: 10 min before blood sampling** Intervention group: 3.39 ± 0.62 Control group: 3.57 ± 0.76 *p* = .098.	**CFS: During blood sampling (*p* = .000)** Intervention group: 2.04 ± 0.96 Control group: 3.65 ± 0.67 **CFS: 10 min after blood sampling (*p* = .000)** Intervention group: 0.01 ± 0.10 Control group: 1.84 ± 1.37 **Differences between intervention groups**: ***p* = .000** **Differences between control groups: *p* = .000**	Positive
(Cai et al., 2014)	Depression Anxiety	BDI STAI	At the pretreatment assessment, there were no statistically significant differences between two groups with respect to demographic characteristics. The pretest mean scores revealed no significant differences (*p* > .05) between the two groups in the parameters at baseline.	There was a decrease in the depression (*F*(1,28) ¼ = 18.89; ***p* < .005**) and anxiety (*F*(1, 28) ¼ = 27.11; ***p* < .005**) scores in the humor group from pretest to posttest.	Positive
(Rudnick et al al., 2014)	Depression Anxiety	PANAS	The three study arms showed no significant demographic or clinical (diagnostic and other) differences at baseline (*F* (2, 32) = 1.01, *p* = .375 for age; *F* (2, 32) = 1.16, *p* = .325 for years of education; *F* (2, 32) = 0.80, *p* = .459 for length of psychiatric illness; *F* (2, 32) = 0.680, *p* = .514 for number of psychiatric hospitalizations).	There was no significant difference in attrition between the study arms (FET = 3.77, ***p* = .183**). Reliabilities of all outcome measures were satisfactory.	Neutral
(Tagalidou et al., 2019)	Depression Anxiety	STAI CESD	There were no differences in demographic variables between the intervention and wait list control groups. In addition, two groups had no differences in baseline measures.	The ITT analysis revealed no significant group by time interaction for any outcome: **Depression (*F* (2, 64.86) = 1.18, *p* = .315); Anxiety (*F* (2, 66.53) = 0.56, *p* = .575)** Depression and anxiety showed no effects at all. Post hoc tests did not show significant effects for the training group from pre to post or pre to follow‐up.	Neutral
(Bressington et al., 2019)	Depression Anxiety	DASS	Analysis of participants’ baseline demographic and characteristics revealed no statistically significant differences between the two groups (*p* > .05).	**Depression**: The outcome measure results indicated that the LT group had a statistically greater decrease in depression (DASS21—Depression scale) than the control group from baseline to immediately following the intervention (*B* = −5.123, 95% CI: –9.527 to –0.72; ***p* = .023**). However, there was no significant difference in the change in depression from baseline to 3‐month follow‐up between the two groups (*B* = –2.724; 95% CI: −7.106 to 1.658; ***p* = .223**). **Anxiety**: There were no significant differences in changes in anxiety (DASS21—Anxiety scale) between groups from baseline to the first follow‐up (*B* = −3.256, 95% CI: –7.309 to 0.258; ***p* = .068**) or second follow‐up (*B* = −2.321, 95% CI: –6.458 to 1.816; ***p* = .271**).	Positive
(Shahidi et al., 2011)	Depression	GDS	The baseline outcomes between the three groups were also not significantly different.	The analysis revealed a significant difference in the decrease in depression scores of both the Laughter Yoga and exercise therapy group in comparison to the control group (***p* < .001** and ***p* < .01**, respectively). Laughter therapy: Pretest (mean ± SD): 16 ± 5.3; Posttest (mean ± SD): 10 ± 6.9 Control: Pretest (mean ± SD): 15.2 ± 3.9; Posttest (mean ± SD): 15.2 ± 6.1	Positive
(Kim et al., 2015)	Depression	POMS‐B	Baseline demographic and clinical characteristics for the two groups did not differ at the 5% significance level. **Before Laughter Therapy (*p* = .609)** **Intervention group**: 5.15 ± 4.49 **Control group**: 5.69 ± 3.65	**Pre‐ and Postlaughter therapy**: **Intervention group**: −2.30 ± 3.84 **Control group**: −0.17 ± 3.52 **(*p* = .023, *p* < .05)**	Positive
(Lee et al., 2020)	Depression	BDI	No significant differences were observed when we compared the demographic and clinical characteristics of the laughter and control groups. Furthermore, the preprogramed evaluations revealed that the laughter and control groups had similar levels of stress, depression, and HRQOL.	Relative to the control group, the laughter group exhibited significant improvements in the scores for depression **(*p*¼ .025)**. Furthermore, the laughter group also had a significantly lower incidence of depression based on the scores from Beck's Depression Inventory **(*p* ¼ .047)**. A significant improvement was observed in the laughter group for the mild depression subgroup **(*p* ¼ .009)**.	Neutral
(Genc & Saritas, 2020)	Anxiety	STAI	The results show no statistically significant difference between the patients’ gender, age, marital status, disease diagnosis or level of education in control and intervention groups. The pretest of STAI scores and vital signs were similar in both groups (*p* > .05).	The difference between the mean anxiety scores of the two groups was statistically significant **(*p* = .03, *p* < .05)**. **Intervention group**: 43.36 ± 9.76 **Control group**: 47.13 ± 5.76 The pretest anxiety scores of the individuals in the experimental group decreased from 49.84 ± 8.16 to 43.36 ± 9.76 after watching the video, and the difference between these scores was found to be significant**(*p* < .001)**.	Positive
(Bega et al., 2017)	Depression Anxiety	Neuro‐QoL	No significant differences were observed when we compared the demographic and characteristics of the intervention or control groups. **Anxiety** (*p* = .115) Intervention group: 16 (12, 20) [8, 25] Control group: 20 (16, 25) [9, 33] **Depression** (*p* = .064) Intervention group: 12 (10, 17) [8, 20] Control group: 16 (13, 21) [9, 26]	**Anxiety** (*p* = .380) Pre: 16 (14, 20) [8, 25] Post: 17 (12, 20) [8, 30] Change: −1 (4, 1) [8, 8] **Depression** (*p* = .128) Pre: 13 (11, 20) [8, 26] Post: 12 (9, 16) [8, 24] Change: −1.5 (4, 0) [10, 7] There was no significant improvement in anxiety and depression **(*p* > .05)**.	Positive
(Bennet et al., 2020)	Depression Anxiety	PHQ‐4	There were no differences at baseline on demographic characteristics between groups.	The proportion of patients with self‐reported depressive symptoms changed from 17 (22%) to 16 (20%), in control and from 11 (17%) to 5 (8%) in the intervention, respectively (***p* = .04**). In the control arm, 7 out of the 17 patients with self‐reported depressive symptoms at baseline continued to report depressive symptoms at follow‐up compared to the intervention arm where only 1 of 12 patients continued to report depressive symptoms. No differences were noted between the groups for reported anxiety.	Positive
(Armat et al., 2022)	Depression Anxiety	BDI BAI	Statistical tests showed that the groups were still balanced in terms of age, height, weight, marital status, and educational level.	Depression and anxiety levels were measured at study initiation, week 4, and week 8 in both groups. Results showed a significant difference in the pattern of **depression** (***p* < .001**) and **anxiety** (***p* < .001**) scores within and between groups. The trend of changes in depression score is moderately ascending in the control group, whereas it is sharply descending in the intervention group.	Positive
(Ko et al., 2022 )	Depression Anxiety	CESD BAI	The baseline outcomes between the two groups were also not significantly different.	Outcomes were measured right after the completion of the intervention and 2 weeks later. The levels of acculturative anxiety and depression decreased right after the intervention compared to the baseline, and the effects were sustained after 2 weeks (***p* < .001, *p* < .001,** respectively).	Positive
(Kiyak & Kocoglu, 2021)	Depression Anxiety	STAI BDI	The baseline depression, state anxiety, and trait anxiety mean scores of the **IG** and **CG** were similar.	**Depression** (8.44 ± 6.43) and trait anxiety scores of the IG (45.63 ± 5.05) were lower than the CG (11.57 ± 8.57; 47.93 ± 4.91) and the effect size was small (*d* = 0.35 for depression; *d* = 0.17 for trait anxiety). Group × time interaction was significant for depression (*F* = 99.563, ***p* < .001**) and trait anxiety (*F* = 5.441, ***p* = .021**).	Positive
(Ko & Youn, 2011)	Depression	GDS	There were no significant differences in baseline characteristics between the two groups. **Before laughter therapy,** the GDS scores were as follows: Laughter therapy group: 7.98 ± 3.58 Control groups: 8.08 ± 3.96	**After laughter therapy**, the GDS scores were laughter therapy group:6.94 ± 3.19 (***p* = .027**) control groups: 8.43 ± 3.44(***p* = .422**). ANCOVA, controlling for preexperimental GDS score and other variables, showed statistical significance in the effect of laughter therapy on GDS (***p* = .011**).	Positive
(Heidari et al., 2020)	Depression	Elderly's Depression Questionnaire	There were no significant differences in baseline between the two groups.	The mean scores of depression in the intervention group after LT (*M* = 2.57) were lower than those before the intervention (*M* = 6.87) (95% CI = −5.58 to −3.02) and also the results of independent *t*‐test showed a statistically significant difference before and after the intervention between the two groups (***p* < .001**).	Positive
(Ghodsbin et al., 2015)	Depression Anxiety	GHQ‐28	There were no significant differences in baseline between the two groups.	We found a statistically significant correlation between the laughter therapy program and factors such as anxiety (**Pre: 7.83 ± 4.74, Post: 3.84 ± 2.77, *p* = .001**). However, there was no statistically significant correlation between the laughter therapy and depression (***p* = .069**).	Positive
(Low et al., 2014)	Anxiety	BEAM	There were no differences at baseline in demographic characteristics between groups. IG residents were rated at baseline as having a longer duration of active disengagement and a shorter duration of happy effect.	Over time, there were also significant overall decreases in the duration of anxious. The IG had increased high positive behavior (***p*¼ .017**) and decreased active disengagement (***p*¼ .008**) and angry mood (***p*¼ .033**) in comparison with controls.	Positive
(Brodaty et al., 2014)	Depression Anxiety	CSDD NPI‐NH	There were no differences in baseline measures between the two groups. Assessments were performed at baseline, week 13, and week 26.	**Depression**: Laughter Boss Commitment was associated with higher resident engagement that in turn was associated with decreased depression scores. Similarly, higher management support score predicted higher Laughter Boss Commitment scores, thereby ultimately affecting resident engagement and CSDD scores. The model fit for CSDD was acceptable (***c*2 / df ¼ 1.68, RMSEA ¼ 0.06, CFI ¼ 0.92**). **Anxiety**: Laughter Boss Commitment ratings were associated with resident engagement, which was associated with a decrease in NPI‐NH scores over time. The fit statistics indicated adequate model fit (***c*2 /df ¼ 0.74, RMSEA < 0.001, CFI ¼ 1.00**).	Positive
(Low et al., 2013)	Depression Anxiety	CSDD CMAI	There were no significant differences on demographic characteristics between the groups. Intervention group residents were taking slightly more regular psychotropic medications on average.	Depression decreased over time. The group by time interaction was significant for agitation measured using the CMAI, before and after adjustment for covariates (***p* < .05**). The humor therapy group decreased on the CMAI by 0.17 (95% CI 0.004 to 0.34; ***p* = .045**) points more than controls between baseline and follow‐up.	Positive
(Ozturk & Tezel, 2021)	Depression Anxiety	BSI	There were no differences in baseline measures between the two groups.	Evaluation of the mean scores obtained in BSI subdimensions (i.e., **anxiety, depression**) before and after the intervention showed a significant decrease in the scores of the IG compared with the CG (***p* < .05**). Anxiety (pretest): IG: 1.08(0.47); CG: 1.02 (0.61) Anxiety (posttest) IG: 0.67 (0.50) CG: 0.84 (0.58) ***p* < .001** Depression (pretest) IG: 1.34 (0.57); CG: 1.33 (0.69) Depression (posttest) IG: 0.89 (0.55); CG: 1.25 (0.57) ***p* < .004**	Positive

### 3.1 Study characteristics

These included studies were conducted in Australia (*n* = 3), the USA (*n* = 4), Italy (*n* = 4), Turkey (*n* = 4), South Korea (*n* = 4), Iran (*n* = 4), Israel (*n* = 3), China (*n* = 2), and Germany (*n* = 1). The sample sizes in the 29 studies were between 12 and 398 participants, with a total of 2964 participants. The present review included 26 quantitative studies (Agostini et al., 2014; Cai et al., 2014; Dionigi et al., 2014; Felluga et al., 2016; Goldberg et al., 2014; Kocherov et al., 2016; Kurudirek et al., 2021; Liguori et al., 2016; Meiri et al., 2016; Tagalidou et al., 2019; Shahidi et al., 2011; Bega et al., 2017; Bennett et al., 2020; Genc & Saritas, 2020; Kim et al., 2015; Lee et al., 2020; Armat et al., 2022; Ko et al., 2022; Brodaty et al., 2014; Ghodsbin et al., 2015; Heidari et al., 2020; Kiyak & Kocoglu, 2021; Ko & Youn, 2011; Low et al., 2014; Low et al., 2013; Ozturk & Tezel, 2021), of which one was a quantitative nonrandomized trial, as well as one qualitative study (Tener et al., 2016), and two mixed studies (Bressington et al., 2019; Rudnick et al., 2014). The qualitative study used in‐depth semistructured interview guides to collect data (see Table 2).

Six studies included participants with mental illnesses, such as depressive disorder, dementia, and Parkinson's disease. Nine studies included children undergoing surgery or under anesthesia while six studies analyzed the elderly in nursing homes. Three studies included patients with cancer while a further three investigated retired women, immigrant women, and infertile women. Bennett et al. (2020) analyzed hemodialysis patients, while Ozturk & Tezel (2021) included first‐year nursing students in their study (see Table 3).

### 3.2 Methodological quality

The included studies were found to be of moderate quality (*n* = 6), moderately high quality (*n* = 16), and high quality (*n* = 7). The quality ratings of studies based on the MMAT criteria are provided in Table 2. A list of the specific quality criteria for each study is also provided in Table 4.

**TABLE 4:** List of quality evaluation grades (*N* = 29).

		Study
Category of study designs	Methodological quality criteria	Tener 2016
Screening questions (for all types)	S1. Are there clear research questions?	√
	S2. Do the collected data allow to address the research questions?	√
	*Further appraisal may not be feasible or appropriate when the answer is “No” or “Can't tell” to one or both screening questions*.	
**1. Qualitative**	1.1. Is the qualitative approach appropriate to answer the research question?	√
	1.2. Are the qualitative data collection methods adequate to address the research question?	√
	1.3. Are the findings adequately derived from the data?	√
	1.4. Is the interpretation of results sufficiently substantiated by data?	√
	1.5. Is there coherence between qualitative data sources, collection, analysis, and interpretation?	√

		Study											
**Category of study designs**	**Methodological quality criteria**	Meiri 2016	Dionigi 2014	Liguori 2016	Goldberg 2014	Felluga 2016	Kocherov 2016	Agostini 2014	Kurudirek 2021	Cai 2014	Kim 2015	Genc 2020	Bega 2017
Screening questions (for all types)	S1. Are there clear research questions?	√	√	√	√	√	√	√	√	√	√	√	√
	S2. Do the collected data allow to address the research questions?	√	√	√	√	√	√	√	√	√	√	√	√
	*Further appraisal may not be feasible or appropriate when the answer is “No” or “Can't tell” to one or both screening questions*.												
**2.Quantitative randomized controlled trials**	2.1. Is randomization appropriately performed?	√	√	√	√	√	√				√	√	
	2.2. Are the groups comparable at baseline?	√	√	√	√	√	√	√	√	√	√	√	√
	2.3. Are there complete outcome data?	√	√	√	√	√	√	√	√	√	√	√	√
	2.4. Are outcome assessors blinded to the intervention provided?				√								√
	2.5 Did the participants adhere to the assigned intervention?	√	√	√	√	√	√	√	√	√	√	√	√

		Study												
**Category of study designs**	**Methodological quality criteria**	Armat 2022	Ko 2022	Kiyak 2021	Shahidi 2011	Ko 2011	Heidari 2020	Ghodsbin 2015	Low 2014	Brodaty 2014	Low 2013	Tagalidou 2019	Ozturk 2021	Bennett 2020
Screening questions (for all types)	S1. Are there clear research questions?	√	√	√	√	√	√	√	√	√	√	√	√	√
	S2. Do the collected data allow to address the research questions?	√	√	√	√	√	√	√	√	√	√	√	√	√
	*Further appraisal may not be feasible or appropriate when the answer is “No” or “Can't tell” to one or both screening questions*.													
**2. Quantitative randomized controlled trials**	2.1. Is randomization appropriately performed?	√	√	√			√	√	√		√	√	√	√
	2.2. Are the groups comparable at baseline?	√	√	√	√	√	√	√	√	√	√	√	√	√
	2.3. Are there complete outcome data?	√	√	√	√	√	√	√	√	√	√	√	√	√
	2.4. Are outcome assessors blinded to the intervention provided?		√				√		√		√			
	2.5 Did the participants adhere to the assigned intervention?	√	√	√	√	√	√	√	√	√	√	√	√	√

		Study
**Category of studydesigns**	**Methodological quality criteria**	Lee 2020
Screening questions (for all types)	S1. Are there clear research questions?	√
	S2. Do the collected data allow to address the research questions?	√
	*Further appraisal may not be feasible or appropriate when the answer is “No” or “Can't tell” to one or both screening questions*.	
**3. Quantitative nonrandomized**	3.1. Are the participants representative of the target population?	√
	3.2. Are measurements appropriate regarding both the outcome and intervention (or exposure)?	√
	3.3. Are there complete outcome data?	√
	3.4. Are the confounders accounted for in the design and analysis?	√
	3.5. During the study period, is the intervention administered (or exposure occurred) as intended?	√

		Study			
**Category of study designs**	**Methodological quality criteria**	/	/	/	/
Screening questions (for all types)	S1. Are there clear research questions?				
	S2. Do the collected data allow to address the research questions?				
	*Further appraisal may not be feasible or appropriate when the answer is “No” or “Can't tell” to one or both screening questions*.				
**4. Quantitative descriptive**	4.1. Is the sampling strategy relevant to address the research question?				
	4.2. Is the sample representative of the target population?				
	4.3. Are the measurements appropriate?				
	4.4. Is the risk of nonresponse bias low?				
	4.5. Is the statistical analysis appropriate to answer the research question?				

		Study	
**Category of study designs**	**Methodological quality criteria**	Rudnick 2014	Bressington 2019
Screening questions (for all types)	S1. Are there clear research questions?	√	√
	S2. Do the collected data allow to address the research questions?	√	√
	*Further appraisal may not be feasible or appropriate when the answer is “No” or “Can't tell” to one or both screening questions*.		
**5. Mixed methods**	5.1. Is there an adequate rationale for using a mixed methods design to address the research question?	√	√
	5.2. Are the different components of the study effectively integrated to answer the research question?	√	√
	5.3. Are the outputs of the integration of qualitative and quantitative components adequately interpreted?	√	√
	5.4. Are divergences and inconsistencies between quantitative and qualitative results adequately addressed?	√	√
	5.5. Do the different components of the study adhere to the quality criteria of each tradition of the methods involved?		

### 3.3 The epidemiology of depression or anxiety in different populations

Six articles exclusively explored the impact of humor therapy on depression while 12 studies examined its effect on anxiety and 11 investigated its effects on both depression and anxiety. The findings of these studies were summarized in Table 4. Notably, depression and anxiety are often comorbid with other illnesses or experiences. For instance, Agostini et al. (2014) reported a high incidence of anxiety symptoms in children scheduled for surgery, with approximately 75% of children receiving anesthesia experiencing severe anxiety or pain. Similarly, Genç and Saritas (2020), Liguori et al. (2016), and Kocherov et al. (2016) found that 60‒80% of children and 40‒75% of adults undergoing surgical procedures experience high levels of preoperative anxiety. Brodaty et al. (2014) investigated the residents of 17 old‐age homes in Sydney, Australia, and found that 25‒40% of the residents suffered from depression. Two studies (Heidari et al., 2020; Ko & Youn, 2011) reported an increased prevalence of mental disability in elderly populations, particularly in nursing homes, where depressive symptoms increased by up to 20‒30% as the numbers of elderly residents increased. Kim et al. (2015) reported that cancer and tumor patients have a high degree of psychological distress, and 20‒40% of such people were simultaneously diagnosed with depression and anxiety, necessitating timely treatment and intervention. Bega et al. (2017) and Bennett et al. (2020) studied depressive symptoms in patients with Parkinson's disease and hemodialysis, where the incidence of depression was 17% and 39.3%, respectively. Furthermore, in a recent study on stress among married immigrant women (MIV) in South Korea, Ko et al. (2022) found that acculturation stress resulted in higher levels of depression and anxiety in this population, reaching 31.2%.

### 3.4 Research tools

The included studies used various scales to evaluate the effectiveness of humor therapy on anxiety or depression. Six articles (21%) used the State‐Trait Anxiety Inventory (STAI) (Spielberger et al., 1983), a Likert‐type scale in which 20 questions are set to assess state and trait anxiety levels, with scores ranging from 1 to 4 for each question. Total scores on the state and trait anxiety subscales ranged from 20 to 80, with higher scores indicating higher anxiety levels. Four articles (14%) used the Beck Depression Inventory (BDI) (Beck et al., 1961). The BDI is a 21‐item self‐reporting scale consisting of emotional, cognitive, somatic, and motivational components to measure changes in the severity of depressive symptoms. Each answer is scored between 0 and 3 (From no symptoms to severe symptoms). The scale yields a total score of 0−63. Four articles (14%) used the modified Yale Preoperative Anxiety Scale (M‐YPAS) (Kain et al., 1995). This was used to assess the level of anxiety in children undergoing anesthesia induction. M‐YPAS consists of 22 items that address the activity, emotional expression, arousal status, vocalization, and parental need in young children. Children's anxiety levels are assessed by evaluating their behavior in five different areas, with higher scores indicating greater anxiety. Kain et al. (1995) confirmed the validity of this assessment. Three articles (10%) utilized the Cohen‐Mansfield Agitation Inventory (CMAI) (Cohen‐Mansfield et al., 1989), which includes 29 types of agitated behaviors, with each behavior rated on a scale of 1 to 7. The higher the score, the greater the likelihood that the patient will exhibit agitated behaviors. The Beck Anxiety Inventory (BAI) (Beck et al., 1988; Beck et al., 1996) was used in two articles. This consists of 21 questions related to general anxiety symptoms. Each item is scored from 0 to 3, and the total score ranges from 0 to 63, with higher scores indicating more severe anxiety symptoms. Two studies used the Cornell Scale for Depression in Dementia (CSDD) (Alexopoulos et al., 1988), a scale designed to assess depression in dementia patients; the scores range between 0 and 28, with higher scores indicating higher levels of depression in dementia patients. Two studies used the Geriatric Depression Scale (GDS) (Yesavage et al., 1982) to assess depressive mood, with a score of 0 to 9 indicating no depression, 10 to 19 indicating moderate depression, and 20 or more indicating major depression. One article used the Neuropsychiatric Inventory‐Nursing Home version (NPI‐NH) (Cummings et al., 1994), assessing the frequency and severity of delusions, irritability, anxiety, irritability, and hyperexcitability. In this scale, the scores range from 0 to 144, with higher scores indicating an increased number of symptoms and a higher frequency of occurrence. One article used the Chinese version of the Depression Anxiety Stress Scale (DASS−21) (Lovibond & Lovibond, 1995) while another study used the German Center for Epidemiological Studies Depression Scale Revised (CESD‐R) (Hautzinger et al., 2012). Most of these research tools were screening scales, not diagnostic tools, so the results must be interpreted cautiously.

### 3.5 Study findings

In general, the studies included in this review found that humor therapy, a kind of nondrug therapy, has received increasing attention. It was used progressively in clinical practice to improve the symptoms of depression or anxiety in all types of people. The results of 27 included studies showed that humor techniques such as humor therapy, clown intervention, and laughter therapy/yoga could reduce depression or anxiety. However, two articles did not show a significant effect of humor therapy on depression or anxiety and remained neutral. Of course, humor therapy has some limitations, and there will be still plenty of room for future research.

#### 3.5.1 The scope and content of humor therapy

Humor therapy is usually divided into “spontaneous” (humorous) and “simulated” (nonhumorous) laughter. The practice of inducing spontaneous laughter mainly included comedy videos, standup comedies, role‐playing such as medical clowns, etc. “Simulated” laughter therapy usually involves clapping, dancing, and elements that do not involve laughter, such as laughter yoga. Table 4 lists interventions for depression or anxiety. The 29 studies focused on three types of humor therapy interventions, including humor therapy, medical clowning, and laughter therapy. Humor therapy interventions included showing participants comedy videos or movies. Sometimes, Laughter Bosses and Elder Clowns interact with them through music, jokes, slapstick, or simply conversation in both one‐on‐one or in groups. Medical clowns entertained children in various ways, such as interrupting, soap bubbles, magic tricks and puppets, pantomime techniques, prestidigitation, juggling, and improvisation. Armat et al. (2022) concluded that laughter therapy could expand the sternum, exercise breathing ability, stimulate the brain to generate a happy mood, and then relax the entire body.

#### 3.5.2 The effects of three kinds of intervention on depression or anxiety

##### Humor therapy

Seven studies demonstrated the effect of humor therapy on depression or anxiety. These studies were comparable to baseline measures and did not have statistically significant differences in demographics or preintervention. Three studies had shown that humor therapy improved depression and anxiety symptoms in elderly seniors in nursing homes; the intervention process was conducted by the “Laughter Boss” or the “Elder Clown” (Brodaty et al., 2014; Low et al., 2014; Low et al., 2013). In patients with mental disorders, humor therapy had a different therapeutic effect. Through humor skills training, Cai et al. (2014) found that symptoms of depression and anxiety improved significantly in 15 subjects, and BDI and STAI scores were significantly reduced (*p* < .005). However, Rudnick et al. (2014) and Tagalidou et al. (2019) both demonstrated that standup comedy training and humorous games did not improve depression and anxiety, particularly major depression. As a result, both studies were neutral with respect to humor therapy. Genc and Saritas (2020) discussed the effect of watching comedy videos on anxiety symptoms in cancer patients, and the results demonstrated that humor therapy can effectively relieve anxiety (*p* < .001). This review revealed that the actual physical condition of the patients will determine the timing of each intervention. In most cases, it will take at least 9–12 humor training sessions.

##### Medical clowns

Multiple studies had shown that medical clowns were popular among pediatric patients (Van Venrooij & Barnhoorn, 2017). In this review, nine articles addressed the role of medical clowns in the field of medicine, especially pediatrics (Dionigi et al., 2014; Felluga et al., 2016; Goldberg et al., 2014; Kocherov et al., 2016; Liguori et al., 2016; Meiri et al., 2016; Tener et al., 2016; Agostini et al., 2014; Kurudirek et al., 2021). These studies highlighted the sources of anxiety in children who receive clinical treatment and explored in more detail the effect of medical clowns on the reduction of anxiety in children, of course, at a comparable baseline. Separation from parents, fear of unfamiliar environments or people, pain, and fear of medical procedures can all contribute to hospitalized children being more prone to anxiety and stress. Eight studies found that medical clowning significantly relieved anxiety in children, with significant reductions in VAS, m‐YPAS, STAI, CAPS, and CFS scores (*p* < .05). Tener et al. (2016) interviewed participants in an in‐depth semistructured interview, they described the significance of the clown retrospectively and how the clown projected onto their perception of the hospital, the examination, and their personal narrative. Research suggested that the presence of medical clowns throughout the medical process, while largely eliminating pain for children and families, may even be a positive empowering experience, reshaping perceptions of the hospital experience. Clown doctors normally performed their duties when a doctor is treating a child and the duration of the intervention was relatively short. Several studies have also emphasized the relevance of clown interventions in reducing preoperative anxiety and emotional responses in both children and their parents (Agostini et al., 2014; Dionigi et al., 2014; Meiri et al., 2016; Tener et al., 2016; Kocherov et al., 2016). This could be because parents’ emotions, behavior, and health all played roles in their children's psychological experiences. Stress and anxiety from parents can easily be passed on to their children. In contrast, Agostini et al. (2014) assumed in their studies that the presence of parents could effectively reduce children's pain and anxiety during anesthesia induction.

##### Laughter therapy/yoga

Laughter therapy contained laughter yoga, a complementary intervention since the 1970s (Rosner, 2002). Thirteen articles were introduced to show the effects of laughter therapy on depression or anxiety (Bressington et al., 2019; Shahidi et al., 2011; Armat et al., 2022; Bega et al., 2017; Bennett et al., 2020; Ghodsbin et al., 2015; Heidari et al., 2020; Kim et al., 2015; Kiyak & Kocoglu, 2021; Ko & Youn, 2011; Ko et al., 2022; Lee et al., 2020; Ozturk & Tezel, 2021). Laughter therapy generally involved laughter, deep breathing, stretching, meditation, and music‐related activities (chorusing, body movement, and dancing). This therapy was delivered primarily through group sessions (Lee et al., 2020). In this review, 11 studies had shown that laughter therapy improved depression, including depression, cancer, hemodialysis, retired women, immigrant women, infertile women, nursing home seniors, and freshmen. With the same baseline, there were significant differences in DASS, CDS, POMS‐B, PHQ‐4, BDI, CESD, and BSI scores between the pre and postlaughter therapy interventions. (*p* < .05). However, Ghodsbin et al. (2015) indicated that the intervention group did not improve depression among 36 nursing home seniors after a six‐week laughter therapy program. The study further explained that the difference could be due to the short duration of the study and timely changes in depressive mood. If we could continue the study over a longer period of time, we might see significant changes in depression scores. Again, Bega et al. (2017) confirmed that laughter therapy did not improve depression and anxiety in Parkinson's patients, but significantly improved subjects’ ability to function in daily life. Eight studies discussed the effects of laughter therapy on anxiety, and most of the studies found that laughter therapy obviously improved participants’ anxiety, but Bressington et al. (2019) confirmed that implementing a group‐based laughter yoga intervention did not improve anxiety in depressed patients. According to Rudnick et al. (2014), the positive effect of laughter therapy on stress and depression may be due to the psycho‐neuro‐endocrine‐immune stress response mechanism. Armat et al. (2022) concluded that laughter therapy could expand the sternum, exercise breathing ability, stimulate the brain to generate a happy mood, and then relax the entire body. Only physical and mental relaxation can maintain positive emotions while reducing depression, anxiety, and stress.

#### 3.5.3 Challenges for the future of humor therapy

Humor was influenced by personal factors such as personality, age, and gender characteristics, where men were more likely to use humor than women, and there may be large differences in the emphasis on humor use (Schweikart, 2020). Clinically, medical personnel should fully consider the environmental factors that affect humor in the implementation of humor therapy, such as stress, degree of perceptual difficulty, preonset physical symptoms, number of negative attitudes, the severity of illness, pain, executive ability, etc. In summary, we should pay attention to the personality differences of the patients to select the best treatment plan.

The therapeutic effects of humor were widely accepted and had persisted for centuries, but there was no consensus on the definition of humor, the type of intervention, or the best way to assess the effects. There is limited empirical evidence to support mediation mechanisms for the positive effects of humor, and the lack of empirical evidence limits the acceptance and use of humor therapy by clinical medical personnel (Savag et al., 2017). Brain imaging provides a means to study the mediating mechanisms of the positive effects of humor. However, until now, there have been no studies involving the neurological effects of humor interventions.

## 4 DISCUSSION

This integrative review compiled the results of previously published randomized controlled trials and qualitative and mixed studies related to the topic of humor therapy and systematically synthesized the narrative summaries of a range of CAM‐based multivariate humor therapies in patients with anxiety and depression. In general, the results of the review showed that humor therapy has considerable developmental prospects and advantages in the treatment of anxiety and depression. It can be actively put into practice to reduce the adverse effects of mild anxiety and depression in patients.

### 4.1 The importance of mental and psychological rehabilitation

Depression and anxiety disorders are prevalent throughout the world and also represent a focus of attention in the field of mental illness. The World Health Organization emphasizes strengthening mental health care in its 2022 World Report on Mental Health. In contrast to traditional rehabilitation, mental illness rehabilitation focuses on reducing psychopathology. Therefore, the integration of new forms of treatment to improve patients’ happiness, life satisfaction, self‐esteem, and other positive emotions is crucial.

### 4.2 The universality of the use of humor therapy

Humor therapy is increasingly recognized as a cost‐effective, safe, and efficient intervention to enhance physical and mental health, as well as social well‐being. A recent study found that approximately 11.8% of children in the United States prefer complementary and alternative medicine. Among the nonpharmacological approaches, clown therapy has gained widespread popularity (Dionigi et al., 2014). Medical clowns create a more positive atmosphere between the medical team and patients by conveying their sense of humor through whimsical antics, comedy, and improvisation (Gomberg et al., 2020). Going through surgery and the use of syringes is painful and frightening for children and, furthermore, the COVID‐19 pandemic had also forced children worldwide to get vaccinated. Therefore, helping children reduce anxiety and fear of syringes is also important for future research, and the collaboration of medical clowns and nurses should address this issue. At the same time, nurses are also in charge of vaccinating and injecting; their humor skills will also positively impact this process. This type of intervention can meet the needs of individual children, has both short‐ and long‐term effects, and is widely used.

Dr. Madan Kataria in India proposed the concept of laughter yoga as part of laughter therapy in 1994. This was a type of yoga that incorporates yogic breathing, meditation, and relaxation. Unlike other types of yoga, laughter yoga practice does not necessitate special training facilities or professional yoga equipment, nor does it necessitate professional inspection or supervision due to its simple movements, safety, and low intensity. Practitioners can learn and practice alone quickly, at a low cost, with high patient participation and ease of compliance (Miles et al., 2016).

### 4.3 Explore the mechanism of humor therapy

Humor is positively correlated with happiness and is a strategy for regulating emotion that is not only a medium for dispersing and transferring negative emotions but also an effective tool for dealing with negative life situations. Humor therapy and the emotional care offered by Chinese medicine coincide. According to traditional Chinese medicine, people have seven emotions: joy, anger, worry, thought, sadness, fear, and shock. “Joy overcomes sorrow” is also proposed in the Yellow Emperor's Inner Canon; a happy mood can eliminate inner depression and annoyance. Considering this, humor and laughter therapies can induce and stimulate laughter, resulting in positive emotions. Of course, traditional Chinese medicine also emphasizes dialectics and tension. As a result, humor intervention should be carried out with regard to the specific situation of individuals and with consideration of the person's internal and external environment, including factors such as age, gender, cultural differences, and sense of humor, amongst others.

Laughter has been shown to promote movement in the respiratory muscles within the chest and abdomen while triggering reflex diaphragmic function through four stages of laryngeal regulation. These stages include the laughter interpulse pause, arytenoid cartilage closure, vocal cord vibration, and arytenoid cartilage opening. In addition, laughter has been found to normalize hypothalamic‐pituitary‐adrenocortical dysfunction. By increasing the frequency of laughter, individuals may reduce symptoms of depression and anxiety by direct improvement of their mood and mental health in response to stressful events, which can be attributed to cortical and subcortical brain regions (Fonzi et al., 2010; Lee et al., 2020).

To summarize, the relationship between humor, laughter, and health has gained increasing attention in healthcare approaches. Physiological models classify laughter into spontaneous and simulated types, both of which have positive effects. In the humor enhancement model, humor induces positive emotions. The pressure‐release model helps individuals relieve stress from their daily lives, while the social model reduces interpersonal conflict and tension, ultimately promoting overall health (Lee et al., 2020).

### 4.4 Strengths and limitations

Although humor therapy is widely used in treating both depression and anxiety, there is no comprehensive summary of its specific interventional methods, duration, efficacy, and patient feedback. This review compiled previously published randomized controlled trials and qualitative and mixed studies related to this topic.

This study was comprehensive regarding the literature search, using publications from five databases. We searched for articles investigating people from different countries, regions, age groups, and disease associations. Several humor therapy interventions were further explored, namely, medical clown therapy, laughter therapy, and laughter yoga, showing that humor interventions can be provided in multiple forms to meet the specific needs of individuals. Furthermore, no adverse effects are associated with humor therapy, and the intervention is relatively easy to implement.

However, this study has several limitations. First, while most of the studies included in the review came to a positive conclusion, it is difficult to generalize the effects of humor therapy on patients with depression or anxiety as different studies used different forms of intervention. According to the literature, humor therapy is effective in patients with mild anxiety and depression, but its efficacy in patients with moderate to severe anxiety and depression needs to be further verified. Second, all of the included studies assessed the effects of depression and anxiety using scales that failed to achieve objective diagnostic results. Third, the inclusion of only one qualitative study is insufficient for analyzing the public's understanding and feelings about humor therapy, resulting in a poor understanding of the potential shortcomings of humor therapy, which may hinder the development of future interventional measures.

## 5 CONCLUSIONS

The study reviewed quantitative, qualitative, and mixed studies on the effects of humor therapy on people with depression or anxiety. The baseline measurements and statistical outcomes of each study were analyzed to explore the therapeutic effects of various interventions, including comedic video and crosstalk‐based humor therapy, medical clowning, and laughter therapy. The review revealed that humor significantly impacts perception, attitude, judgment, and mood, which may directly or indirectly influence physical and mental well‐being. While most studies confirmed the benefits of humor therapy for depression or anxiety, several suggested that the intervention period may have been too short for the full reflection of positive effects. Therefore, additional high‐quality studies are necessary for the further verification of the effects of humor therapy.

### 5.1 Implications

Obstacles are inevitable in the implementation of humor therapy with the most important being the attitude and views of the medical staff towards humor therapy. This is because they are two different teams with different goals. Several studies showed that the work of medical staff may occasionally be hindered due to humor therapy treatment, thus affecting overall patient care and leading to negative views of humor therapy. On the positive side, medical personnel may also consider that humor therapy can relieve pain, reduce the negative effects of treatment, and have a positive impact on overall patient recovery. Therefore, medical personnel need to be consulted before the implementation of humor therapy to achieve the maximum effects of the therapy.

The future success of humor therapy depends on its pricing and the effectiveness of its services. If humor therapy is applied to special groups such as the elderly and cancer patients, the inclusion of humor therapy in the national healthcare system is a powerful tool for its promotion.

Humor therapy can be combined with specific technological applications and treatments in some cases and situations. Humor therapy often cannot be fully implemented because some hospitals prohibit clown doctors from entering operating theaters and interactions were also severely curtailed in specific situations such as during the COVID‐19 pandemic, restricting the use of the therapy. Thus, the use of apps, video recordings, and live guidance can compensate for these drawbacks. Technology is the best way to reach more patients.

Future research should use widely accepted definitions of humor and effective assessment tools to try to assess and test the effects of humor interventions based on neurobiological effects and laboratory marker tests to better understand how humor therapy affects mental health.

## AUTHOR CONTRIBUTIONS

Jindan Zhang, Xiaotu Zhang, and Yidan Wang conceived the study. Jindan Zhang, Yidan Wang, and Sixuan Li screened the studies and extracted data. Xuefeng Sun, Zihan Qu, and Xiaotu Zhang did the quality assessment. Xuefeng Sun wrote the first draft of the manuscript. All authors revised this draft. All authors read and approved the final version.

## CONFLICT OF INTEREST STATEMENT

The authors declare no conflict of interest.

### PEER REVIEW

The peer review history for this article is available at https://publons.com/publon/10.1002/brb3.3108.

## Data Availability

All figure and data analysis are fully available without restriction.
